# Transcatheter Management of Congenital and Acquired Venous Lesions in Children

**DOI:** 10.3390/jcm15145499

**Published:** 2026-07-14

**Authors:** Utku Pamuk, Emine Azak, Yasemin Özdemir Şahan, Cansu Çetin Şentürk, Ayşe Ünal Yüksekgönül, Oguzhan Dogan, Hazım Alper Gursu, İbrahim İlker Çetin

**Affiliations:** 1 Department of Pediatric Cardiology, Ankara Yıldırım Beyazıt University, Ankara 06800, Türkiye; iicetin@hotmail.com; 2 Department of Pediatric Cardiology, University of Health Sciences, Ankara 06800, Türkiye; azakemi@gmail.com (E.A.); hagursu@yahoo.com.tr (H.A.G.); 3 Department of Pediatric Cardiology, Ankara Bilkent City Hospital, Ankara 06800, Türkiye; dr.yaseminozdemir@gmail.com (Y.Ö.Ş.); cansucetin3567@gmail.com (C.Ç.Ş.); dr.unalays@gmail.com (A.Ü.Y.); 4 Division of Pediatric Cardiology, Department of Pediatrics, Mehmet Akif İnan Training and Research Hospital, Şanlıurfa 63300, Türkiye; oguzhandogan32@hotmail.com

**Keywords:** transcatheter intervention, pediatric, venous abnormalities, portosystemic shunt, pulmonary venous obstruction, glenn circulation, veno-venous collaterals

## Abstract

**Background:** Congenital and acquired venous abnormalities in children represent a heterogeneous group of conditions associated with significant clinical morbidity, including systemic desaturation, hepatic dysfunction, and life-threatening hemodynamic compromise. Evidence regarding transcatheter management across this diverse spectrum remains limited and largely confined to small, lesion-specific series. This study aimed to evaluate the feasibility, procedural, and clinical outcomes of transcatheter venous interventions in a heterogeneous pediatric population. **Methods:** This retrospective single-center study included all consecutive pediatric patients (median age: 9 years; range: 2 days–17 years) who underwent a total of 28 transcatheter procedures for congenital and acquired venous abnormalities between January 2022 and September 2025. Lesions included congenital portosystemic shunts (*n* = 4), systemic venous stenoses (*n* = 2), obstructed pulmonary venous pathways (*n* = 3), and veno-venous collaterals or azygos/hemiazygos continuation associated with Glenn circulation (*n* = 9). Procedural strategies were tailored according to anatomical characteristics, and outcomes were assessed in terms of technical success, procedural complications, and lesion-specific clinical and hemodynamic improvement. **Results:** A total of 28 transcatheter procedures were performed in 18 patients. The median procedure duration was 120 min (range, 35–235 min), and the median follow-up duration was 13.5 months (range, 0.6–35 months). During follow-up, two patients required reintervention for restenosis of previously treated lesions, whereas five additional procedures were performed because of newly developed veno-venous collaterals. Transcatheter interventions resulted in lesion-specific clinical and hemodynamic improvements, including reductions in serum ammonia levels, relief of venous obstruction, and improvements in oxygen saturation. One patient with complex pulmonary venous obstruction died following subsequent surgery despite initial hemodynamic improvement. **Conclusions:** In this single-center experience, transcatheter venous interventions were feasible and were associated with favorable immediate and mid-term clinical and hemodynamic outcomes in children with diverse venous abnormalities. Given the small sample size, heterogeneous patient population, and retrospective design, these findings should be considered preliminary. Larger prospective multicenter studies are warranted to better define optimal patient selection, timing of intervention, and long-term outcomes.

## 1. Introduction

Congenital and acquired venous abnormalities in children represent a heterogeneous group of conditions that may lead to significant clinical consequences, including systemic desaturation, hepatic dysfunction, venous hypertension, pulmonary hypertension, or life-threatening cardiorespiratory compromise [1,2,3]. These lesions encompass a broad anatomical spectrum—ranging from congenital portosystemic shunts (CPSS) and systemic venous stenoses to obstructed pulmonary venous pathways and right-to-left shunting lesions associated with Glenn circulation, such as persistent azygos or hemiazygos continuation and veno-venous collaterals. Because of their rarity and diversity, these conditions are often described only in isolated case reports or small, lesion-specific series, and the optimal timing and technique of intervention remain subjects of ongoing clinical discussion.

Over the past two decades, transcatheter therapy has emerged as an essential component of management for selected pediatric venous lesions. Endovascular closure of portosystemic shunts can reverse metabolic complications and prevent long-term hepatic sequelae; balloon angioplasty and stenting of systemic venous obstructions provide rapid decompression and restore venous return; and stenting of obstructed pulmonary venous pathways may offer temporary stabilization or serve as a bridge to surgery in critically ill infants [4,5]. In patients with single-ventricle physiology, catheter-based occlusion of azygos continuation or veno-venous collaterals has become a key strategy to address refractory cyanosis and improve candidacy for Fontan completion [6].

Despite increasing use of these techniques, comprehensive reports integrating the full spectrum of pediatric venous lesions remain limited, as most studies focus on individual lesion types. Although these conditions differ in anatomy, pathophysiology, and clinical presentation, they share common interventional principles aimed at restoring venous drainage, relieving obstruction, or eliminating pathological venous communications. Given the rarity of each individual lesion, a descriptive overview encompassing multiple venous pathologies may provide practical insights into contemporary transcatheter management. The present study describes a single-center experience with transcatheter treatment of congenital and acquired venous lesions in children, focusing on lesion-specific technical approaches, procedural outcomes, and clinical outcomes across this heterogeneous spectrum.

## 2. Materials and Methods

Between January 2022 and September 2025, the study population comprised all consecutive children who underwent transcatheter intervention for clinically significant congenital or acquired venous lesions at our tertiary center. Patients were considered for transcatheter intervention following multidisciplinary evaluation by the institutional pediatric cardiology and cardiovascular surgery teams, based on lesion anatomy, hemodynamic significance, clinical status, and the anticipated feasibility of catheter-based treatment. These lesions included congenital portosystemic shunts associated with biochemical abnormalities (e.g., hyperammonemia or hepatic lesions), systemic venous obstruction involving the superior vena cava (SVC) or inferior vena cava (IVC), obstructed pulmonary venous pathways, and veno-venous collaterals or persistent azygos/hemiazygos continuation causing desaturation in patients with Glenn physiology. The analysis included only transcatheter venous interventions. Accordingly, ductal stenting performed in one patient with pulmonary atresia was excluded from the total intervention count.

Procedural outcomes were assessed using predefined technical, hemodynamic, and clinical criteria. Technical success was defined as successful completion of the intended transcatheter intervention without major intraprocedural complications or the need for emergency surgery. Hemodynamic success was defined as lesion-specific objective improvement, including reduction in a measured pressure gradient or restoration of venous flow. Clinical success was defined as improvement in the principal clinical or biochemical consequence of the lesion, including reduction in serum ammonia, improvement in systemic oxygen saturation, resolution of pericardial effusion, successful central venous access, or stabilization permitting subsequent surgery [7,8]. Procedural failure was defined as inability to complete the intended intervention, occurrence of a major intraprocedural complication, or the need for emergency surgical conversion.

Clinical follow-up included outpatient assessment, echocardiography, cross-sectional imaging when indicated, and biochemical evaluation in patients with CPSS. Follow-up focused on lesion-specific clinical and hemodynamic outcomes, need for reintervention, and survival.

All procedures were performed under general anesthesia with systemic heparinization and under fluoroscopic guidance in the pediatric cardiac catheterization laboratory. Periprocedural antibiotic prophylaxis was administered when considered clinically appropriate. Vascular access was selected according to lesion anatomy, most commonly through the right internal jugular vein; in one patient, bilateral jugular venous access was required to establish a controlled veno-venous loop. In patients undergoing closure of CPSS or occlusion of azygos/hemiazygos continuation or veno-venous collaterals, balloon occlusion testing was performed to evaluate intraportal and pulmonary artery pressure responses before definitive device deployment.

### Statistical Analysis

Descriptive statistical analyses were performed to summarize patient demographics, lesion characteristics, procedural variables, and clinical outcomes. Categorical variables were reported as frequency (*n*) and percentage (%), while continuous variables were presented as median and range. All analyses were conducted using SPSS Statistics version 26.0 (IBM Corp., Armonk, NY, USA).

## 3. Results

A total of 18 pediatric patients underwent 28 transcatheter procedures for congenital and acquired venous lesions (Table 1). The median age was 9 years (range, 2 days–17 years), and the cohort included 8 males and 10 females. The median procedure duration was 120 min (range, 35–235 min), and the median follow-up duration was 13.5 months (range, 0.6–35 months). Patients were categorized into four lesion groups: congenital portosystemic shunts (CPSS, *n* = 4), systemic venous stenoses involving the superior or inferior vena cava (*n* = 2), obstructed pulmonary venous pathways (*n* = 3), and lesions associated with Glenn physiology, including persistent azygos/hemiazygos continuation or veno-venous collaterals (*n* = 9). All intended transcatheter interventions were successfully completed without major intraprocedural complications. One patient with Scimitar vein stenosis died following subsequent surgical repair despite initial hemodynamic improvement after transcatheter intervention. During follow-up, two patients required reintervention for restenosis of previously treated lesions, whereas additional procedures were performed as part of planned staged treatment or to treat newly developed veno-venous collaterals. No device-related complications were observed.

### 3.1. Congenital Portosystemic Shunts

Four patients with congenital portosystemic shunts—three with Abernethy type II malformations and one with a patent ductus venosus—underwent transcatheter closure. Patients were symptomatic, presenting with hyperammonemia, hepatic adenomas, or complications related to abnormal portal venous circulation. Pre-procedural imaging and balloon occlusion testing demonstrated adequately developed intrahepatic portal venous structures and stable portal pressures, enabling safe endovascular intervention. Access was obtained via the jugular vein in three patients and the femoral vein in one.

**Figure 1:**
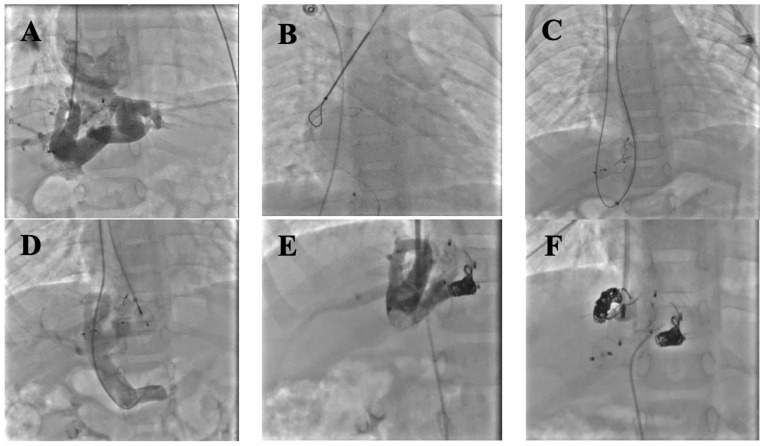
Staged transjugular closure of multiple congenital portosystemic shunts using low-profile occlusion devices, vascular plugs, coils, and a jugular–jugular veno-venous loop technique. (**A**) Angiographic image after deployment of a Konar MFO device and an Amplatzer Duct Occluder II device during the first two procedures, demonstrating multiple residual portosystemic shunts. (**B**) Establishment of a jugular–jugular veno-venous loop by snaring a guidewire advanced through a separate portosystemic shunt. (**C**) Advancement of a long sheath over the established veno-venous loop. (**D**) Deployment of a Ceraflex vascular plug into the target large portosystemic shunt. (**E**) Coil embolization to eliminate residual peri-device flow. (**F**) Final angiographic image demonstrating coil embolization of the residual right-sided shunt and deployment of an additional Ceraflex vascular plug within the remaining central shunt.

The first patient was a 2.5-year-old boy with persistent hyperammonemia who required four staged procedures for complete closure of multiple congenital portosystemic shunts. During the first two sessions, two distinct tortuous shunts were occluded using low-profile occlusion devices (Konar MFO and Amplatzer Duct Occluder II) (Figure 1A). During the third stage, a controlled jugular–jugular veno-venous loop was established to facilitate catheterization of an otherwise inaccessible shunt, allowing deployment of a Ceraflex vascular plug (Figure 1B–D). During the final stage, residual peri-device flow and a residual right-sided shunt were eliminated using coil embolization, while the remaining large central shunt was occluded with another Ceraflex vascular plug (Figure 1E,F).

The other two patients underwent successful single-session closure. One was a 17-year-old girl with Down syndrome and recurrent hyperammonemia who underwent successful transfemoral closure using an Amplatzer septal occluder. The other was a 17-year-old girl with tricuspid atresia after Fontan palliation who developed hepatic adenomas. Following multidisciplinary evaluation, the shunt was successfully closed by transcatheter deployment of a Ceraflex vascular plug through the Fontan pathway.

The final patient was a one-month-old infant with a patent ductus venosus who presented with persistent respiratory distress and progressive hyperammonemia. Owing to progressive metabolic deterioration, transcatheter closure was performed using a Ceraflex vascular plug. This resulted in rapid biochemical improvement, with reductions in serum ammonia and liver enzyme levels, and the patient was discharged without complications one week later.

Overall, transcatheter closure was associated with reductions in serum ammonia levels and lesion-specific clinical improvement in all four patients during follow-up, without any procedure-related complications.

### 3.2. Central Venous Obstructions

**Figure 2:**
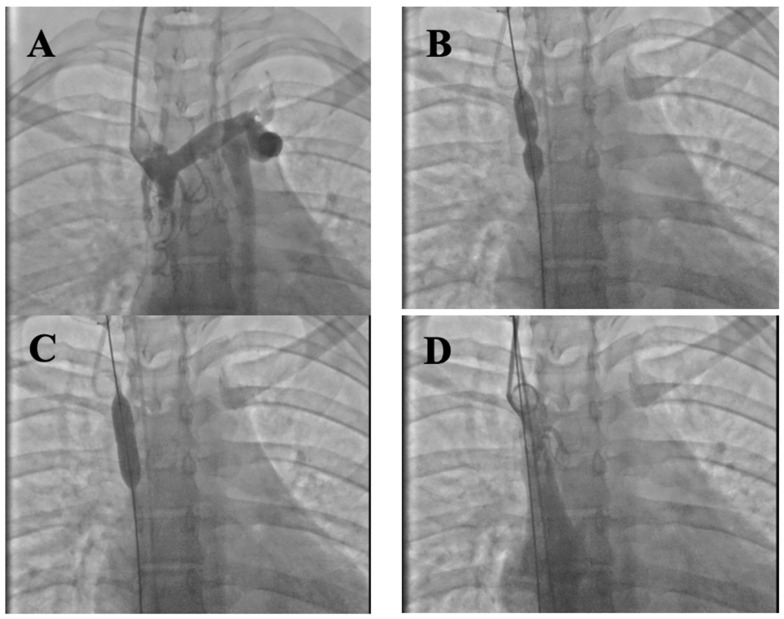
Balloon angioplasty of severe superior vena cava stenosis in a child with systemic venous obstruction. (**A**) Angiographic image demonstrating severe superior vena cava stenosis with collateral drainage through a dilated hemiazygos vein. (**B**) Inflation of a non-compliant balloon across the stenotic segment demonstrating marked balloon indentation. (**C**) Complete balloon expansion following relief of the stenosis. (**D**) Final angiographic image demonstrating restoration of superior vena cava patency after balloon angioplasty.

Two patients underwent balloon angioplasty for clinically significant systemic venous stenosis. The first patient was a 17-year-old girl receiving chemotherapy for Ewing sarcoma whose repeated attempts at central venous port placement had failed because of near-total SVC obstruction with collateral drainage through a dilated hemiazygos vein. Transfemoral balloon angioplasty restored SVC patency, allowing successful port placement (Figure 2A–D). At 12-month follow-up, the superior vena cava remained patent without evidence of recurrent stenosis.

The remaining patient was a 13-year-old boy with a history of renal transplantation and recurrent pericardial effusions who had residual IVC–right atrium junctional stenosis following previous surgery for a membranous IVC web, as well as a separate SVC–right atrium junctional stenosis with azygos collateral drainage. Balloon angioplasty resulted in resolution of the pericardial effusion; however, recurrent pericardial effusion one year later required repeat balloon angioplasty of both stenotic segments. The patient remained clinically stable without recurrence of pericardial effusion during 2 years of follow-up.

Balloon angioplasty restored central venous patency and was associated with sustained clinical improvement in both patients. One patient required repeat intervention because of restenosis, whereas the other remained free of recurrent stenosis throughout follow-up. No procedure-related complications were observed.

### 3.3. Stenting of Obstructed Pulmonary Venous Pathways

Three patients with obstructed pulmonary venous pathways underwent transcatheter stenting. In two patients, the procedure was performed as an emergency stabilization strategy because of severe clinical and hemodynamic deterioration. In the third patient, transcatheter intervention was selected after multidisciplinary discussion because the patient’s critical condition and low body weight precluded immediate surgical repair.

A 4-month-old girl with Scimitar syndrome presented with severe cardiorespiratory compromise and critical Scimitar vein stenosis. Balloon angioplasty alone failed to adequately relieve the obstruction, necessitating stent implantation, which reduced the pressure gradient from 39 to 16 mmHg and resulted in transient hemodynamic improvement despite a residual gradient. The patient subsequently underwent surgical repair but died in the postoperative intensive care unit.

Another patient was a 1.5-month-old boy with obstructed supracardiac total anomalous pulmonary venous return (TAPVC) who underwent emergency catheterization because of clinical deterioration. Balloon angioplasty alone resulted in significant elastic recoil, necessitating stent implantation in the vertical vein. Balloon atrial septostomy was additionally performed because of restrictive atrial communication and hemodynamic compromise. The patient subsequently underwent successful surgical repair and remained well during 31 months of follow-up.

**Figure 3:**
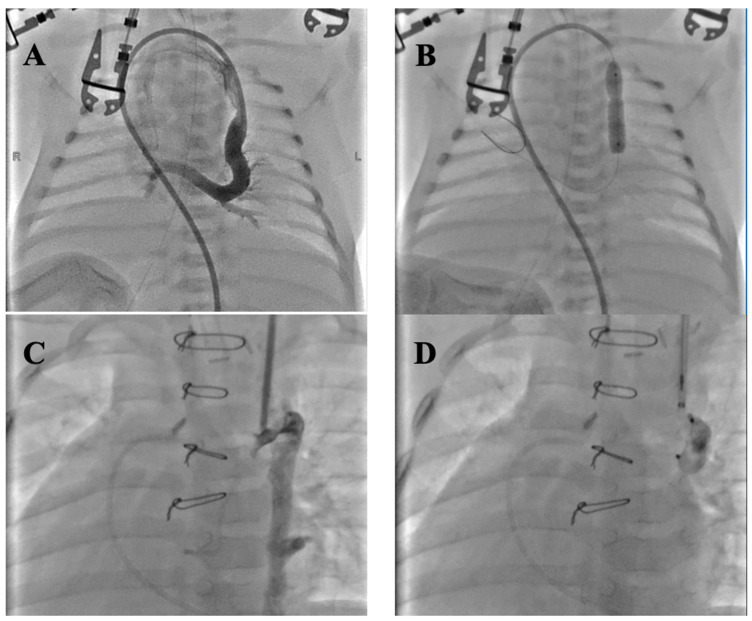
Representative transcatheter interventions for obstructed pulmonary venous pathways and veno-venous collateral circulation in children with complex congenital heart disease. (**A**) Angiography after predilatation with a coronary balloon showing persistent critical stenosis of the vertical vein before stent implantation. (**B**) Stent implantation across the stenotic vertical vein, restoring pulmonary venous drainage. (**C**) Angiographic image demonstrating a patent hemiazygos continuation following bidirectional Glenn circulation. (**D**) Transcatheter occlusion of the hemiazygos continuation using an Amplatzer Duct Occluder II device.

The youngest patient was a 2-day-old, 2 kg neonate with right atrial isomerism, pulmonary atresia, and obstructed supracardiac TAPVC. Transcatheter stenting of the vertical vein was performed after predilation of the stenotic segment (Figure 3A,B). Two weeks later, ductal stenting was performed via carotid arterial access to maintain pulmonary blood flow. During follow-up, in-stent restenosis of the vertical vein resulted in recurrent desaturation and required reintervention 1.5 months later. Balloon angioplasty reduced the pressure gradient from 13 to 3 mmHg and improved oxygen saturation from 76% to 90%. The patient remained alive at 2 months of follow-up.

Transcatheter stenting provided immediate hemodynamic stabilization in all three patients. Two patients remained alive at 2 and 31 months of follow-up, whereas one patient with complex Scimitar syndrome died following subsequent surgical repair despite initial hemodynamic improvement after transcatheter intervention. One patient required reintervention because of in-stent restenosis, and no procedure-related complications were observed.

### 3.4. Transcatheter Occlusion of Azygos/Hemiazygos Continuation and Veno-Venous Collaterals in Pre-Fontan Circulation

Nine patients with single-ventricle physiology and prior bidirectional Glenn circulation underwent a total of 14 transcatheter procedures because of progressive cyanosis caused by persistent azygos/hemiazygos continuation or large veno-venous collaterals. Contrast echocardiography demonstrated early contrast entry into the atria, suggesting systemic venous bypass. Catheter angiography confirmed persistent azygos/hemiazygos continuation or large veno-venous collaterals as the source of right-to-left shunting.

Persistent azygos or hemiazygos continuation was identified in three patients (Figure 3C,D). In one patient, the channel had been intentionally left patent during the initial operation because of elevated Glenn pressures, whereas surgical details were unavailable in the remaining two patients. Balloon occlusion testing demonstrated no significant increase in pulmonary artery pressure, and all azygos/hemiazygos continuations were successfully occluded using Amplatzer Duct Occluder II devices.

Large veno-venous collaterals originating from the brachiocephalic vein or superior vena cava and draining into the atria were identified in seven patients. One patient also had concomitant azygos continuation. These vessels were successfully occluded using Amplatzer Piccolo occluders, Amplatzer Duct Occluder II devices, or a Ceraflex vascular plug according to vessel anatomy.

Transcatheter occlusion resulted in immediate improvement in systemic oxygen saturation in all patients. During follow-up, additional transcatheter procedures were performed in patients who developed new veno-venous collaterals, whereas no procedure-related complications were observed.

## 4. Discussion

Transcatheter management of pediatric venous abnormalities remains challenging because of the heterogeneity of underlying lesions, variable clinical presentation, and the lack of standardized treatment algorithms. Most published studies have focused on individual lesion types, limiting the applicability of their findings to broader clinical practice. In this context, we present a single-center experience encompassing a broad spectrum of congenital and acquired pediatric venous lesions. Our findings suggest that, with careful anatomical assessment, multidisciplinary patient selection, and lesion-specific procedural planning, transcatheter intervention is a feasible treatment strategy across diverse pediatric venous conditions, including critically ill neonates and patients with complex anatomy.

In this single-center cohort, 28 transcatheter procedures were performed in 18 pediatric patients with a broad spectrum of congenital and acquired venous lesions. Lesion-specific clinical and hemodynamic improvement was observed following intervention, including normalization of metabolic abnormalities after congenital portosystemic shunt closure, restoration of venous patency after angioplasty or stent implantation, and improved systemic oxygen saturation following occlusion of right-to-left venous shunts. In critically ill infants with obstructed pulmonary venous return, transcatheter intervention also served as a bridge to subsequent definitive management.

Transcatheter closure has become an established treatment option for carefully selected patients with CPSS, particularly in the presence of hyperammonemia, hepatic lesions, pulmonary hypertension, or other complications related to abnormal portal venous drainage [9,10,11]. Previous series have reported high technical success and favorable clinical outcomes, although treatment strategy should be individualized according to shunt anatomy and the development of the intrahepatic portal venous system, and staged transcatheter closure has been recommended for patients with multiple or complex CPSS to reduce the risk of portal hypertension and allow gradual adaptation of the intrahepatic portal venous circulation [12]. Our patient with multiple Abernethy type II shunts required four staged procedures, illustrating that exceptionally complex shunt anatomy may necessitate multiple staged interventions.

Congenital portosystemic shunts have also been reported in patients with Fontan circulation, where they may contribute to cyanosis, heart failure, and unfavorable long-term outcomes [13,14]. Transcatheter closure of congenital CPSS in patients with Fontan circulation has rarely been reported [14]. Percutaneous treatment has also been described for acquired portosystemic shunts developing after Fontan surgery [15]. Our experience expands the limited published experience and suggests that transcatheter closure can be successfully performed in carefully selected patients following meticulous procedural planning.

Superior vena cava obstruction in children is most commonly an acquired condition related to previous central venous catheterization or cardiothoracic surgery, whereas IVC stenosis is considerably less common and is usually associated with prior surgical interventions [16,17,18,19]. Balloon angioplasty is generally considered the preferred initial endovascular treatment; however, restenosis frequently necessitates subsequent stent implantation, particularly in central venous stenoses [4,20]. Our findings are broadly consistent with these reports, as one patient required repeat intervention because of restenosis; however, both patients were ultimately managed successfully without permanent stent implantation.

Because systemic veins are highly compliant and can often be safely dilated to relatively large diameters, non-compliant balloons were selected to achieve controlled expansion of the stenotic segments [21]. Balloon angioplasty successfully restored venous patency in both patients. One patient remained free of recurrent SVC stenosis at 12-month follow-up, whereas the patient with combined SVC and IVC stenoses required repeat balloon angioplasty because of restenosis but subsequently remained clinically stable during 2 years of follow-up. In this patient, stent implantation was intentionally avoided because of the proximity of the IVC stenosis to the hepatic venous inflow, where permanent stenting could potentially compromise hepatic venous drainage. These findings suggest that, although stent implantation is frequently reported for recurrent central venous stenosis, balloon angioplasty alone may achieve satisfactory medium-term outcomes in carefully selected pediatric patients.

Obstructed pulmonary venous pathways constitute a life-threatening condition requiring urgent restoration of pulmonary venous drainage. In patients who deteriorate rapidly before surgery, transcatheter stenting has been increasingly used as a bridge to definitive surgical repair [22,23,24]. Scimitar vein stenosis is similarly uncommon, and published experience with transcatheter stent therapy remains extremely limited [25]. In our series, one patient with obstructed supracardiac TAPVC and another with severe Scimitar vein stenosis underwent urgent transcatheter stent implantation because of rapid clinical deterioration. In both patients, balloon angioplasty alone failed to provide adequate relief, necessitating stent implantation. In the patient with obstructed supracardiac TAPVC, balloon atrial septostomy was additionally performed because of restrictive atrial communication and persistent hemodynamic compromise. In the patient with Scimitar syndrome, despite incomplete stent expansion, meaningful hemodynamic improvement was achieved, suggesting that complete angiographic relief may not always be necessary to obtain clinically relevant stabilization before surgery. Our findings are consistent with previous reports demonstrating that transcatheter intervention can provide effective temporary preoperative stabilization in critically ill infants [26].

A different clinical scenario was encountered in our 2 kg neonate with right atrial isomerism, pulmonary atresia, and obstructed supracardiac TAPVC. Because immediate surgery was considered prohibitively high risk after multidisciplinary discussion, staged transcatheter intervention was undertaken as a bridge to definitive surgical repair and represented the only feasible therapeutic strategy at that time. Sequential vertical vein stenting, ductal stenting, and subsequent balloon angioplasty for in-stent restenosis maintained adequate pulmonary venous drainage and pulmonary blood flow, allowing continued clinical stabilization until definitive surgical repair becomes feasible. This approach is in keeping with previous reports describing transcatheter palliation in highly selected neonates when immediate surgery is not feasible [23].

Taken together, these cases support previous reports that transcatheter intervention can provide effective temporary stabilization in critically ill infants with obstructed pulmonary venous drainage until definitive surgical repair becomes feasible.

Veno-venous collaterals and persistent azygos or hemiazygos continuation are well-recognized causes of progressive cyanosis after bidirectional Glenn circulation, primarily due to right-to-left shunting and reduced effective pulmonary blood flow [27]. To avoid adverse hemodynamic consequences, we routinely performed balloon occlusion testing before occluding persistent azygos continuations and large veno-venous collaterals to confirm that pulmonary artery pressures remained within an acceptable range [28]. Following transcatheter occlusion, all patients in our cohort experienced an immediate improvement in systemic oxygen saturation, aligning with the established literature [29]. However, because the underlying hemodynamic stimulus often persists, veno-venous collateral formation remains a dynamic process. Consistent with previous reports, five additional catheter interventions were required during follow-up because of newly developed collateral pathways, highlighting the importance of continued surveillance after Glenn circulation [27].

Beyond lesion-specific outcomes, our experience also highlights several technical considerations that may facilitate transcatheter management of complex pediatric venous abnormalities. Procedural strategies were tailored according to lesion anatomy rather than a uniform approach, and several procedures required modification of conventional techniques because of complex anatomy, small patient size, or limited vascular access. In patients with multiple congenital portosystemic shunts, staged transcatheter closure allowed gradual elimination of complex shunt anatomy, while a controlled jugular–jugular veno-venous loop enabled catheterization of an otherwise inaccessible large shunt. Tortuous collateral vessels were successfully accessed using microcatheters, allowing selective coil embolization. In patients with systemic venous stenosis, satisfactory luminal expansion was achieved using non-compliant balloon angioplasty without the need for permanent stent implantation. In the low-weight neonate with obstructed supracardiac TAPVC, predilatation with a coronary balloon facilitated subsequent passage and deployment of the stent across the critically narrowed vertical vein. Collectively, these technical adaptations, derived from our single-center experience, may improve procedural feasibility and safety in anatomically challenging pediatric patients.

This study has several limitations. It is a retrospective single-center study with a relatively small sample size and marked heterogeneity of the underlying venous lesions. This heterogeneity limited the generalizability of the findings and required lesion-specific assessment of technical and clinical outcomes. In addition, follow-up duration was variable and relatively short in some patients, and the absence of a comparator group precluded direct comparison with alternative treatment strategies. Despite these limitations, our series provides a comprehensive overview of transcatheter management across a broad spectrum of pediatric venous abnormalities, reflecting real-world clinical practice. Our findings suggest that transcatheter interventions are feasible and may represent clinically useful, minimally invasive treatment options, either as definitive therapy or as a bridge to surgery in selected patients. Further prospective multicenter studies with larger cohorts are needed to better define patient selection, procedural strategies, and long-term outcomes.

## 5. Conclusions

Transcatheter intervention can be successfully applied across a broad spectrum of congenital and acquired pediatric venous lesions when treatment strategies are individualized according to lesion anatomy and clinical presentation. Careful procedural planning and continued follow-up are essential, particularly in patients at risk of restenosis or recurrent collateral formation.

## Figures and Tables

**Table 1:** Demographic and Clinical Characteristics of Patients.

Case	Age	Sex	Diagnosis	Intervention	Device Used	Follow-Up	Outcomes
1	2.5 y	M	Multiple CPSS	Transjugular multistage closure	Ceraflex VP; Konar MFO; ADO II; coils	20 mo	NH_3_ 161 → 30
2	17 y	F	CPSS	Transfemoral closure	ASO	10 mo	NH_3_ 48 → 6
3	17 y 4 m	F	CPSS, TA	Transjugular closure	Ceraflex VP	12 mo	NH_3_ 138 → 29
4	1 m	M	Patent ductus venosus	Transjugular closure	Ceraflex VP	13 mo	NH_3_ 103 → 11
5	17 y	F	SVC stenosis	Balloon angioplasty	Z-Med balloon	12 mo	Patent SVC
6	13 y	M	SVC—IVC stenosis	Balloon angioplasty (2 sessions)	Z-Med balloons	24 mo	No pericardial effusion
7	4 m	F	Scimitar vein stenosis	Balloon angioplasty + stenting	Tyshak balloon; Formula stent	<1 mo	Deceased *
8	1.5 m	M	Vertical vein stenosis, TAPVC	Balloon angioplasty + stenting	VACS II balloon; Formula stent	31 mo	SpO_2_ 78 → 89%; PV grad. 16 → 10 mmHg
9	2 days	F	Vertical vein stenosis, TAPVC, RAI, PA	Stenting + repeat balloon angioplasty	Bentley stent	2 mo	SpO_2_ 76 → 90%; PV grad. 13 → 3 mmHg
10	10 y	M	V-V collaterals, hypoplastic RV	Transjugular occlusion	ADO II; coils	7 mo	SpO_2_ 79 → 91%
11	2 y 8 m	F	V-V collaterals, u-AVSD	Transjugular occlusion	APO (×2); coils	14 mo	SpO_2_ 70 → 82%
12	6 y 8 m	F	V-V collateral, c-TGA, VSD, PA	Transjugular occlusion	Ceraflex VP	10 mo	SpO_2_ 71 → 81%
13	10 y	M	Azygos continuation, hypoplastic RV	Transjugular occlusion	ADO II	14 mo	SpO_2_ 72 → 85%
14	15 y	F	V-V collaterals, u-AVSD	Transjugular occlusion	ADO II; APO (×3)	35 mo	SpO_2_ 69 → 81%
15	11 m	M	Azygos continuation, u-AVSD	Transjugular occlusion	ADO II	31 mo	SpO_2_ 65 → 75%
16	9 y	F	V-V collateral, hypoplastic RV	Transjugular occlusion	ADO II	24 mo	SpO_2_ 72 → 80%
17	12 y 7 m	F	V-V collateral, hypoplastic RV	Transjugular occlusion	ADO II	8 mo	SpO_2_ 68 → 79%
18	9 y 9 m	M	Azygos continuation, V-V collateral, DORV, VSD, PS	Transjugular occlusion	ADO II (×2)	16 mo	SpO_2_ 75 → 85%

Abbreviations and manufacturer information: CPSS—congenital portosystemic shunt; Ceraflex VP—Ceraflex Vascular Plug (Lifetech Scientific, Shenzhen, China); Konar MFO—Konar Multifunctional Occluder (Lifetech Scientific, Shenzhen, China); ADO II—Amplatzer Duct Occluder II (Abbott, Plymouth, MN, USA); ASO—Amplatzer Septal Occluder (Abbott, Plymouth, MN, USA); TA—tricuspid atresia; SVC—superior vena cava; IVC—inferior vena cava; Z-Med balloon—Z-Med balloon catheter (NuMED Inc., Hopkinton, NY, USA); Tyshak balloon—Tyshak balloon catheter (NuMED Inc., Hopkinton, NY, USA); Formula stent (Cook Medical, Bloomington, IN, USA); TAPVC—total anomalous pulmonary venous connection; VACS II balloon—VACS II balloon catheter (OSYPKA AG, Rheinfelden, Germany); PV grad.—pulmonary venous pressure gradient; RAI—right atrial isomerism; PA—pulmonary atresia; Bentley stent (Bentley InnoMed GmbH, Hechingen, Germany); RV—right ventricle; u-AVSD—unbalanced atrioventricular septal defect; APO—Amplatzer Piccolo Occluder (Abbott, Plymouth, MN, USA); V-V—veno-venous; c-TGA—congenitally corrected transposition of great arteries; VSD—ventricular septal defect; DORV—double outlet right ventricle; PS—pulmonary stenosis. * The patient died following subsequent surgical repair, unrelated to the transcatheter intervention.

## Data Availability

The data presented in this study are available from the corresponding author upon reasonable request. The data are not publicly available due to patient privacy and institutional data-protection restrictions.
